# Hepatoprotective effect of Crocus sativus (saffron) petals extract against acetaminophen toxicity in male Wistar rats

**Published:** 2014

**Authors:** Arash Omidi, Narges Riahinia, Mohammad Bagher Montazer Torbati, Mohammad-Ali Behdani

**Affiliations:** 1 *Department of Animal Health Management, School of Veterinary Medicine, Shiraz University, I. R. Iran*; 2 *Department of Animal Sciences, Agriculture Faculty, Birjand University, Birjand, I. R. Iran*; 3 *Saffron Research Group, Birjand University, Birjand, I. R. Iran*

**Keywords:** *Acetaminophen(APAP)*, *Crocus* sativus (CS), *Flowers*, *Hepatotoxicity*, *Rats*

## Abstract

**Objectives:** Acetaminophen (APAP) toxicity is known to be common and potentially fatal. This study aims to investigate the protective effects of hydroalcoholic extract, remaining from *Crocus sativus* petals (CSP) against APAP-induced hepatotoxicity by measuring the blood parameters and studying the histopathology of liver in male rats.

**Materials and Methods:** Wister rats (24) were randomly assigned into four groups including: I) healthy, receiving normal saline; II) Intoxicated, receiving only APAP (600 mg/kg); III) pre-treated with low dose of CSP (10 mg /kg) and receiving APAP (600 mg/kg); IV) pre-treated with high dose of CSP (20 mg/kg) and receiving APAP (600 mg/kg).

**Results:** The APAP treatment resulted in higher levels of alanine aminotransferase (ALT), aspartate aminotransferase (AST), and bilirubin, along with lower total protein and albumin concentration than the control group. The administration of CSP with a dose of 20 mg/kg was found to result in lower levels of AST, ALT and bilirubin, with a significant higher concentration of total protein and albumin. The histopathological results regarding liver pathology, revealed sever conditions including cell swelling, severe inflammation and necrosis in APAP-exposed rats, which was quiet contrasting compared to the control group. The pre-treated rats with low doses of ‍CSP showed hydropic degeneration with mild necrosis in centrilobular areas of the liver, while the same subjects with high doses of ‍CSP appeared to have only mild hepatocyte degeneration.

**Conclusions:** Doses of 20 mg/kg of CSP ameliorates APAP–induced acute liver injury in rats. It was concluded that the antioxidant property of CSP resulted in reducing the oxidative stress complications of toxic levels of APAP in intoxicated rats.

## Introduction

The liver is a vital organ that regulates a wide variety of biochemical processes and also plays an important role in the metabolism of carbohydrates, proteins and lipids. Detoxification of potentially toxic chemicals, drugs and environmental contaminants is mainly related to the function of an applicable and healthy liver (Bechmann et al., 2012 ▶). Hepatotoxicity or liver injury, induced by drugs, is one of the major causes of liver diseases (Lee, 2003 ▶). Acetaminophen (APAP or paracetamol or N-acetyl-p-aminophenol), widely used as an analgesic and antipyretic drug for many years (Shah et al., 2011 ▶), has become one of the most commonly taken drugs that often leads to an overdose in various countries (Bunchorntavakul and Reddy, 2013 ▶). APAP is metabolized to N-acetyl-p-benzoquinone imine (NAPQI) by cytochrome P450 enzymes in the liver which is normally produced in small amounts at therapeutic doses of APAP, but in case of overdose, massive loads of NAPQI is produced (Tan et al., 2008 ▶).The depletion of glutathione causes severe damages to the liver when it conjugates with this excess NAPQI. Depletion of glutathione increases the susceptibility of cells towards oxidative stress (Hinson et al., 2010 ▶).

*Crocus sativus* (CS) or saffron has a long history of being used as a spice, medicine, and yellow dye (Melnyk et al., 2010 ▶).CS may have anti-cancer (Abdullaev, 2002 ▶), anti-depressant (Schmidt et al., 2007 ▶), nerve protection (Tamaddon fard et al., 2014 ▶), anti-inflammatory (Nam et al., 2010 ▶) and antioxidant properties (Trujillo et al., 2013 ▶).The growing period of CS is mostly winter and spring, marking it as a popular crop in arid and semi-arid regions of Iran (Azizi-Zohan et al., 2008 ▶). CS petal stands (CSP) as the main by-product of CS processing, since it costs plenty less in comparison to the stigma of CS. Various beneficial properties of CSP has been reported including, antidepressant effects, practical in the treatment of mild-to-moderate depression (Moshiri et al., 2006 ▶), a rich source of phenolic compounds, and the potential to be used as a source of dietary flavonoids (Omidi et al., 2013 ▶). A significant correlation between the antioxidant activity of plant materials and the content of phenolic compounds has been demonstrated (Goli et al., 2012 ▶).There are plants that have hepatoprotective properties against APAP toxicity through the mechanism of acting as antioxidants (Olaleye and Rocha, 2008 ▶). Based on the available literatures, the main cause of hepatotoxicity by APAP is oxidative stress induced by NAPQI (Arnaiz, 1995 ▶), and it seems that pre-treatment with CSP extract has not been evaluated in preventing APAP-induced hepatotoxicity. Thus the aim of this study is to investigate the protective effects of CSP against APAP-induced liver toxicity in male Wistar rats.

## Materials and Methods

**Animals**

Twenty-four male Wistar rats with mean body weight of 220±20 g were individually housed in polypropylene cages in standard rat house conditions (22-25ºC on a 12 hours light-dark cycle) and were fed on a pellet diet (Javaneh Khorasan Co, Mashhad, Iran) with free access to tap water. Animals were acclimatized to the laboratory conditions for the duration of seven days before the commencement of the experiment. All the rats were received humane care in accordance to the approval of Institutional ethics committee rules of the Agriculture Faculty of Birjand University.

**Preparing the petal extract**

**Figure 1:**
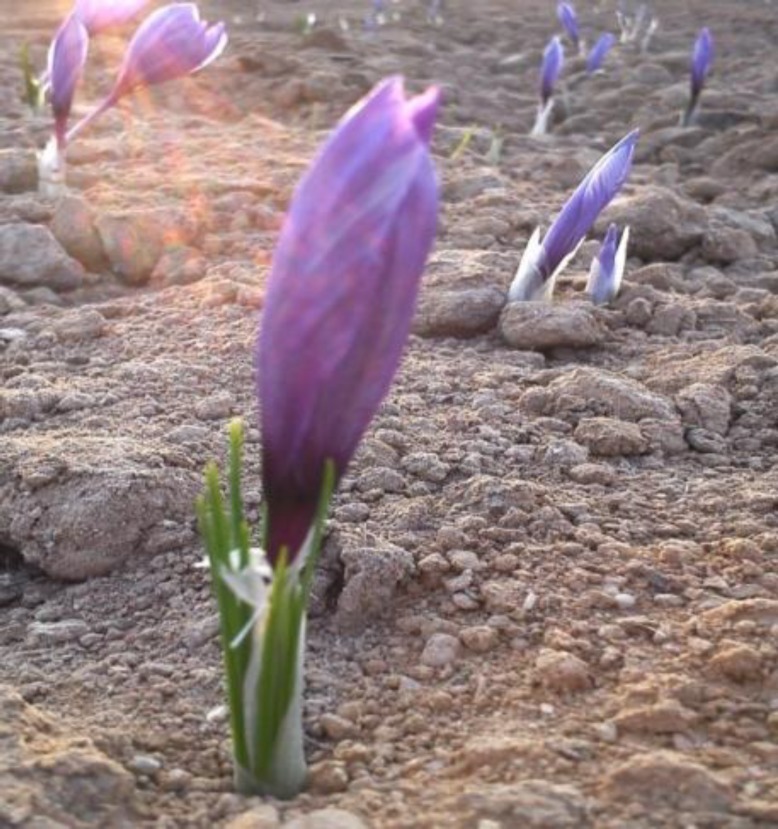
Purple coloured petal of *Crocus sativus*, used in the experiment

CS were collected from Hajiabad Village (Kashmar) in Khorasan-Razavi province, Northeast of Iran, in December 2012 (Figure 1) and had the samples indentified by the Agricultural Faculty of Birjand University in Iran. The voucher number of specimen (No. 2669) was deposited in the herbarium of Birjand University as well. To prepare the CSP, the samples were dried in shadow and then pulverized with a grinder (Hamilton Beach Brand, Canada). Hydroalcoholic extract of CSP was prepared by using 50 g of dried powder in 1000 ml of 80% v/v ethanol and shaking for 24 h. The mixture was filtered through a No. 1 Whatman filter paper and thus oven-dried at 40°Cfor 24 hours. The yield (w/w) was 30% after the final powdered extract was weighed and calculated.

**Treatment schedule**

The CSP doses were selected based on the previous reports and pilot studies (Omidi et al., 2013 ▶). Rats were randomly divided into four groups of six and treated as follow: Group I served as the normal control and received normal daily saline for seven days; Group II served as APAP intoxicated and received normal daily saline for six days and APAP (600 mg/kg) at the seventh day; Group III served as APAP plus10 mg/kg of CSP, and received CSP extract daily for six days but were prescribedAPAP (600 mg/kg) at the seventh day; Group IV served as APAP plus 20 mg/kg of CSP, and received CSP extract daily for six days with APAP (600 mg/kg) prescribed on the seventh day. We dissolved CSP extract in normal saline and had the purepowder of APAP dissolved in 20% alcohol, while administrating all the solutions by oral gavage. After 24 hours of APAP intoxication, rats were euthanized by ether and thus sacrificed.

**Biochemical and histopathological analysis**

Blood sample was collected by cardiac puncture and had the serums separated by centrifugation of the samples at 750×*g* for 15 min at room temperature, storing them at -21°C for further analysis. The concentrations of albumin, total protein, total bilirubin as well as the activities of aspartateaminotransferase (AST), and alanine aminotransferase (ALT), were estimated using Bromocresol green and Biuret method (Pimple et al., 2007 ▶), Malloy and Evelyn method (Malloy and Evelyn, 1949 ▶), and Reitman and Frankel method (Reitman and Frankel, 1957 ▶), respectively by an autoanalyser apparatus (Gesanchem 200, Italy). Diagnostic kits of Pars Azmoon Co. (Iran) were used.The right lobe of the liver was removed and fixed in 10% formalin buffer at room temperature. After fixing the tissue, it was thoroughly washed under running water and dehydrated in ascending grades of ethyl alcohol, cleared afterwards and embedded in soft paraffin. Tissue sections of about 5μm were obtained, and stained by hematoxylin and eosin (H&E), then observed under light microscope (Omidi et al., 2013 ▶).

**Statistical Analysis**

All the tested parameters were subjected to statistical analysis which was done by One-way Analysis of Variance (ANOVA) and had the means compared by Dunnett's comparison. 

## Results

**Figure 2:**
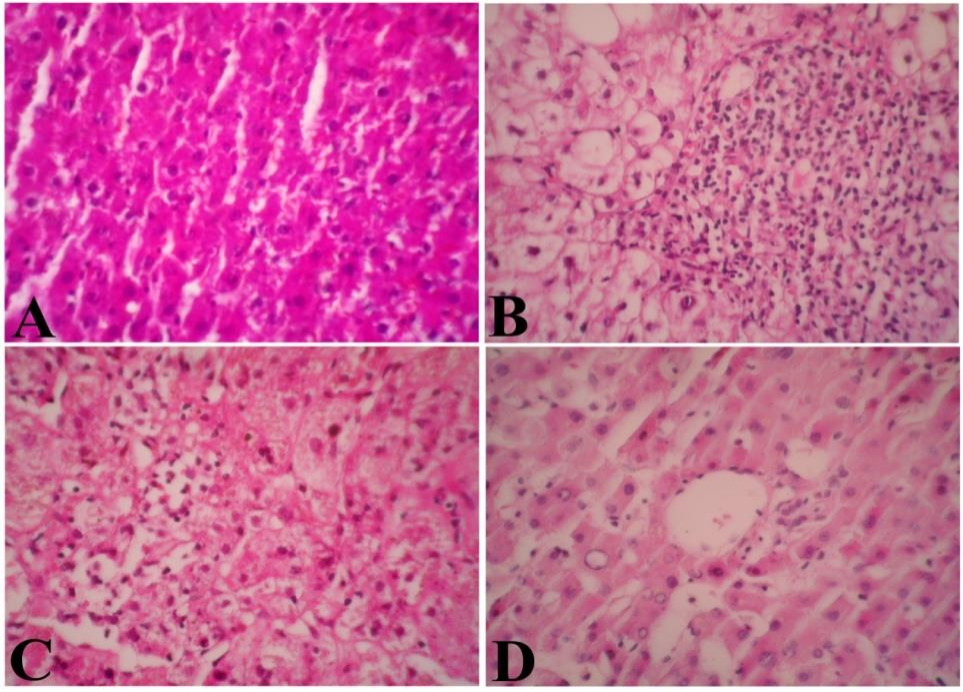
(A) Light microscopic image of the rat liver of the healthy group; normal architecture of the liver was seen (H&E, 40X). (B) Liver of the APAP intoxicated rats; exhibited severe hepatocyte degeneration and necrosis (H&E, 40X). (C) APAP intoxicated rats pre-treated with ‍CSP (10 mg/kg) showed hydropic degeneration with mild necrosis in centrilobular areas of the liver (H&E, 40X). (D) APAP intoxicated rats pre-treated with ‍CSP (20 mg/kg) showed only mild hepatocyte degeneration (H&E).

Administrating APAP to the experimental rats caused severe liver damage with significant increases in the levels of ALT, AST and total bilirubin, as well as a decrease in the levels of total protein and albumin. These results may be due to acute hepatocellular damage and biliary obstruction. Pre-treated rats with CSP extract (20 mg/kg) exhibited nearly normal levels of ALT, AST, total bilirubin, total proteins and albumin. ALT and albumin levels increased and decreased respectively in subjects that received APAP plus 10 mg/kg of CSP (p<0.05). In addition, with administration of CSP (10 mg/kg), the levels of AST, total bilirubin and total protein remained unchanged in comparison to healthy control rats (Table 1). Microscopic studies of the liver samples indicated completely normal and healthy liver tissue structures in the control group (Figure 2-A). Histopathological observations showed severe injuries to the hepatocytes including, cell swelling, severe inflammation and necrosis, in the APAP receiving group, as illustrated in Figure 2-B. The tissue samples in the case of rats, receiving low doses of CSP (10 mg/kg) with APAP, displayed less severe damages compared to the intoxicated rats such as hydropic degeneration with mild necrosis in centrilobular areas (Figure 2-C). Mild degenerative changes were observed as liver injuries, regarding rats with high doses of CSP (20 mg/kg) (Figure 2-D).

**Table 1:** Effects of CSP‎ and APAP on ALT, AST, TB, TP and ALB concentrations in serum of male Wistar rats

**Variables** **(Unit) /Treatment**	**Healthy** ** Control**	**Intoxicated ** **(APAP)**	**APAP+10 mg /kg of CSP**	**APAP+20 mg /kg of CSP**
**ALT (U/l)**	77±3.96	138.7±8.66**	127.7±7.65*	95±6.48
**AST (U/l)**	147.7±9.9	281.9±47.2**	193.6±4.4	154.45±5.5
**TB (mg/dl)**	0.37±0.01	0.54±0.02*	0.47±0.03	0.4±0.02
**TP (g/dl)**	7.1±0.058	6.67±0.1*	6.92±0.1	7.08±0.09
**ALB (g/dl)**	3.20±0.03	2.37±0.06**	2.72±0.12*	3.12±0.07

Values are given as mean ± SEM for six rats in each group.

* Difference is significant at the 0.05 level (two-tailed)

** Difference is significant at the 0.01 level (two-tailed).CSP, *Crocus sativus* petal extract; APAP, acetaminophen; ALT, alanine aminotransferase; AST, aspartate aminotransferase; TB, total bilirubin; TP, total protein; ALB, albumin.

## Discussion

In the present study, APAP-induced hepatotoxicity was evident through biochemical measurements and histopathological findings. APAP significantly increased serum levels of AST, ALT and total bilirubin, whereas notable reduction in the levels of total protein and albumin was observed as well. Damaging ALT and AST, which are found naturally in the liver cells, can result in the release of these enzymes into circulation.Total bilirubin is considered as one of the accurate tests of liver functions since it reflects the ability of this organ to take up and process bilirubin into bile (Arias, 2012 ▶).The normal total protein and albumin levels are associated with appropriate liver function (Barle et al., 2006 ▶), thus the high levels of total bilirubin in the APAP induced treated rats could have been caused by APAP toxicity. The reduction in the total protein and albumin serum, regarding the APAP intoxicated group, might be due to liver damage. According to the histopathological assessment, liver injury in exposed rats was in sharp contrast with the controls (Table 1and Figure 2). 

Severe hepatocyte degeneration and necrosis in the liver of APAP received rats were observed. In APAP intoxication, great amount of NAPQI, extremely toxic to the liver, will be produced. This substance is normally detoxified via conjugation with glutathione to form cysteine and mercapturic acid conjugates (Lu et al., 2013 ▶).

Depletion of hepatic glutathione increases the susceptibility of liver cells to oxidative stress (Tan et al., 2008 ▶; Hinson et al., 2010 ▶; Arnaiz, 1995 ▶).Under conditions of excessive NAPQI formation or hepatic glutathione depletion, NAPQI covalently binds to vital proteins and the lipid bilayer of hepatocyte membranes. However, the present discovery seems to be consistent with other research results, which found massive hepatocyte necrosis, liver failure or death in APAP intoxication (Bunchorntavakul and Reddy, 2013 ▶; Tan et al., 2008 ▶). Many researchers have examined the antioxidant properties of various plants that can support theoxidative defence system, as well as the treatment of liver diseases (Olaleye and Rocha, 2008 ▶). Antioxidants and their potential in health benefits seem to have caught quiet an interest in this field.The antioxidant capacity of CSP is attributed to the presence of flavonoids (Termentzi and Kokkalou, 2008 ▶). In the present study, CSP had a significant effect on the increasing of albumin and total protein levels in serum. Our findings indicate that the administration of CSP extract in two distinct doses almost returned the elevated levels of serum enzymes of APAP, to the normal values ​​(Table 1). 

Histopathological findings also revealed the protective effects of CSP in a dose-dependent manner. The medical uses of CS have a long history, where Avicenna in the Canon of Medicine has described hepatoprotective effects of CS (Hosseinzadeh and Nassiri-Asl, 2013 ▶). In the present time, very few adverse health effects of CS have been demonstrated which were mostly about CS stigma, while toxicity studies are needed for evaluate this substance unfavourable effects (Poma et al., 2012 ▶). However, few studies have shown that the use of CSP at doses of 40 or 75 mg/kg in Wistar rats did not change hematological and biochemical parameters (Omidi et al., 2013 ▶; Babaei et al., 2014 ▶).The mechanism of CSP actions is not profoundly understood. It is likely that the integrity and stability of the cell membrane of liver cells are related to the antioxidant activities of CSP flavonoids.

The findings of the present study showed the notable hepatoprotective activity of CSP on APAP induced hepatotoxicity, which might be associated with its high flavonoid content and antioxidant properties. However, the exact protective mechanism(s) of CSP is unknown. Further investigations with isolated active principles of the plant may further enlighten the use of CSP for hepatoprotective activity.
